# Animation-supported consent for urgent angiography and angioplasty: a service improvement initiative

**DOI:** 10.1136/heartjnl-2019-316227

**Published:** 2020-03-10

**Authors:** David S Wald, Oliver Casey-Gillman, Katrina Comer, Josephine Sarah Mansell, Howie Teo, Kyriacos Mouyis, Matthew Kelham, Fiona Chan, Selda Ahmet, Max Sayers, Vincent McCaughan, Nito Polenio

**Affiliations:** 1 Wolfson Institute of Preventive Medicine, Queen Mary University of London, London, UK; 2 St Bartholomew’s Hospital, Barts Heart Centre, London, UK; 3 Department of Cardiology, Barts Health NHS Trust, London, UK; 4 Department of Cardiology, Newham University Hospital, London, UK; 5 Department of Cardiology, Royal London Hospital, London, UK; 6 Department of Cardiology, North Middlesex University Hospital, London, UK; 7 Department of Cardiology, Whittington Hospital, London, UK; 8 Department of Cardiology, Whipps Cross University Hospital, London, UK; 9 Department of Cardiology, Homerton University Hospital, London, UK; 10 Department of Cardiology, King George Hospital, Ilford, UK; 11 Department of Cardiology, University College Hospital, London, UK

**Keywords:** consent, animation, acute coronary syndrome, angiography, angioplasty

## Abstract

**Objective:**

Patient understanding of angiography and angioplasty is often incomplete at the time of consent. Language barriers and time constraints are significant obstacles, particularly in the urgent setting. We introduced digital animations to support consent and assessed the effect on patient understanding.

**Methods:**

Multi-language animations explaining angiography and angioplasty (www.explainmyprocedure.com/heart) were introduced at nine district hospitals for patients with acute coronary syndrome before urgent transfer to a cardiac centre for their procedure. Reported understanding of the reason for transfer, the procedure, its benefits and risks in 100 consecutive patients were recorded before introduction of the animations into practice (no animation group) and in 100 consecutive patients after their introduction (animation group). Patient understanding in the two groups was compared.

**Results:**

Following introduction, 83/100 patients reported they had watched the animation before inter-hospital transfer (3 declined and 14 were overlooked). The proportions of patients who understood the reason for transfer, the procedure, its benefits and risks in the no animation group were 58%, 38%, 25% and 7% and in the animation group, 85%, 81%, 73% and 61%, respectively. The relative improvement (ratio of proportions) was 1.5 (95% CI 1.2 to 1.8), 2.1 (1.6 to 2.8), 2.9 (2.0 to 4.2) and 8.7 (4.2 to 18.1), respectively (p<0.001 for all comparisons).

**Conclusion:**

Use of animations explaining angiography and angioplasty is feasible before urgent inter-hospital transfer and was associated with substantial improvement in reported understanding of the procedure, its risks and its benefits. The approach is not limited to cardiology and has the potential to be applied to all specialties in medicine.

## Introduction

Patient understanding of angiography and angioplasty is often incomplete at the time of consent.^1 2^ Language barriers and time constraints are significant obstacles, especially in the urgent clinical setting when patients have only 2 to 3 days between presentation with an acute coronary syndrome and their procedure. Effective communication is further complicated by the frequent need to transfer patients between hospitals, so-called inter-hospital transfers, where the recommendation to have a procedure is made by a different doctor to the one performing it, with variable levels of explanation and understanding. Patients are often asked for consent without being given the required time to reflect on the benefits and risks and ask questions.^3^ New approaches are needed to support communication and decision-making.^4^

Multi-language animations describing the benefits, risks and alternatives of angiography and angioplasty (www.explainmyprocedure.com/heart) have been introduced into the elective patient pathway with improvement in understanding before consent.^5^ Patients are sent links to an animation of their procedure weeks beforehand, select their language and watch it at home with their family. Questions are answered at a preoperative appointment 1 week before the procedure and consent then sought on the day by the operator. Implementation of this approach resulted in a threefold increase in reported understanding before consent (30% to 90%) at a UK centre.^5^ Reproducing this in the emergency setting is challenging because of the short time between hospital admission and transfer for a procedure and because patients may not have digital devices or a reliable internet connection in hospital.

We developed internet-free videobooks and a simple implementation policy to introduce animations to support consent among patients transferred for urgent angiography and angioplasty from nine referring hospitals to a specialist cardiac centre. Here, we report the results of a consecutive patient survey of reported understanding before and after introduction of the initiative.

## Methods

**Figure 1:**
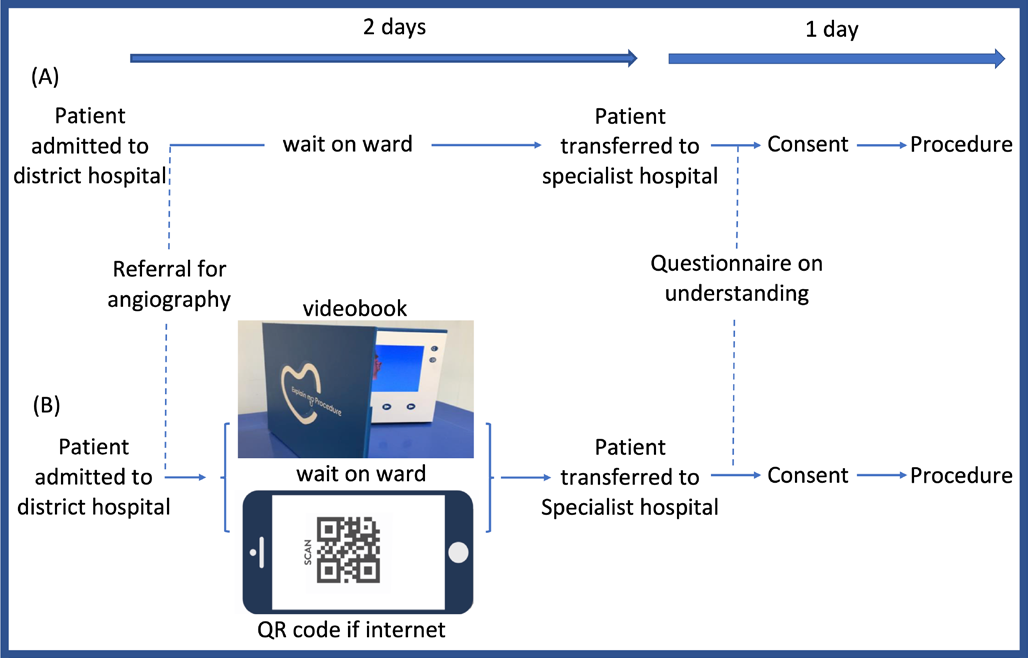
Sequence for 100 patients before (A) and 100 patients after (B) introduction of animation supported consent initiative.

This project was conducted, between July and October 2019, at nine district general hospitals (see list in online supplementary appendix) receiving patients with acute coronary syndromes (non-ST elevation myocardial infarction and unstable angina) and referring them on to a single specialist cardiac hospital (Barts Heart Centre) in London for angiography with possible angioplasty, as urgent inter-hospital inpatient transfers. Figure 1 shows a flow diagram of the pathways for patients before and after introduction of the animation supported consent initiative. We followed the SQUIRE guidance for conducting and reporting quality improvement projects.^6^

10.1136/heartjnl-2019-316227.supp1 Supplementary data

### Animations

Animations explaining angiography and angioplasty, together with the possible benefits and risks of the procedure, were developed according to the NHS England Information Standard^7^ and made available on a website (www.explainmyprocedure.com). Videobooks were manufactured with the animations uploaded, so patients could watch them without internet access if needed. The website and videobook animations were identical and patients were free to view either. Patients were able to select from one of five languages (English, Bengali, Hindi, Turkish and Polish) and could view them multiple times, while awaiting transfer to Barts Heart Centre for their procedure. Patients were involved in the development of the animations to understand aspects of the procedure they found difficult to understand so these could be explained.

### Implementation

A standardised approach to introducing the animations in each of the nine district hospitals was adopted based on five steps: (1) seeking local governance approval for the service development; (2) ‘prescribing’ the animations on the ward round when the procedure was recommended; (3) providing patients with the videobook after the ward round and a leaflet with a weblink and QR code to the animation so this could be reviewed on a personal device and with family (if internet access was available); (4) adding the animation to the inter-hospital transfer checklist completed prior to transfer; (5) adding animation supported consent to junior doctor induction to help sustain the initiative with the arrival of new staff. Implementation was led by the Specialist Registrar in cardiology at each district hospital, who had no role in the development of the animations or collection of data.

### Survey

In the month before implementation of the animations, 100 consecutive patients who had presented with an acute coronary syndrome at any one of the district hospitals were interviewed on arrival at the specialist centre, before their procedure. Patients were asked four questions; whether they understood (1) why they had been transferred to the specialist centre, (2) what the intended procedure involves, (3) the possible benefits of the procedure and (4) the possible risks. Patients were asked to answer using one of three responses (understand completely, understand partly, don’t understand). In the month after introducing the animations into practice, another 100 consecutive inter-hospital transfer patients presenting with an acute coronary syndrome were interviewed on arrival at the specialist centre. Patients were asked the same four questions followed by two more; whether they had watched the video prior to transfer and whether watching the video had helped their understanding of the procedure. Responses were recorded on a questionnaire (online supplementary appendix) by an advanced clinical practitioner who had no role in developing the animations or analysing the results of the audit. Sample size was determined by the time available to complete each audit (1 month) and the number of expected patients in this period (about 100). Assuming an initial level of understanding of about 30%, based on previous work in elective patients,^5^ a study of 200 patients provided in excess of 90% power to show a twofold or greater improvement in reported understanding at a p value of 0.05.

The primary analysis was a comparison of the responses from the patients interviewed after introducing the animations (animation group) with the responses from the patients interviewed before their introduction (no animation group). Secondary analyses were performed, comparing responses from (1) patients in the animation group who watched the animation with those from the no animation group, (2) patients in the animation group who did not watch the animation with those in the no animation group, (3) patients in the animation group who watched the animation with patients in the animation group who did not watch the animation and (4) patients in the animation group who watched the animation in their own language (one of five available) with non–English-speaking patients who were shown it in English (translation in their language unavailable). Responses to the questions were analysed categorically (complete understanding vs partial or no understanding) and ratios calculated by dividing the proportion of positive responses in the animation group by that in the no animation group (with 95% CIs). P values were determined using Fisher’s exact test. Stata V.15 was used for all analyses. The Quality Improvement project was an audit that did not require ethical approval and was registered with the Clinical Effectiveness Board at Barts Health NHS Trust.

## Results

Table 1 shows that patients interviewed before and after introduction of the animations were well matched in terms of age, gender and the proportion of English native speakers, with no statistically significant differences between the groups.

**Table 1:** Characteristics of patients Interviewed before and after introduction of animations to support consent

	Patients interviewed before introduction of animations	Patients interviewed after introduction of animations
n	100	100
No of men	79	79
Age (years)	61	63
Native English speaker	58	63
Bengali	14	11
Turkish	8	5
Hindi/Urdu	5	4
Polish	2	3
Other language	13	14

p>0.1 for all comparisons.

**Figure 2:**
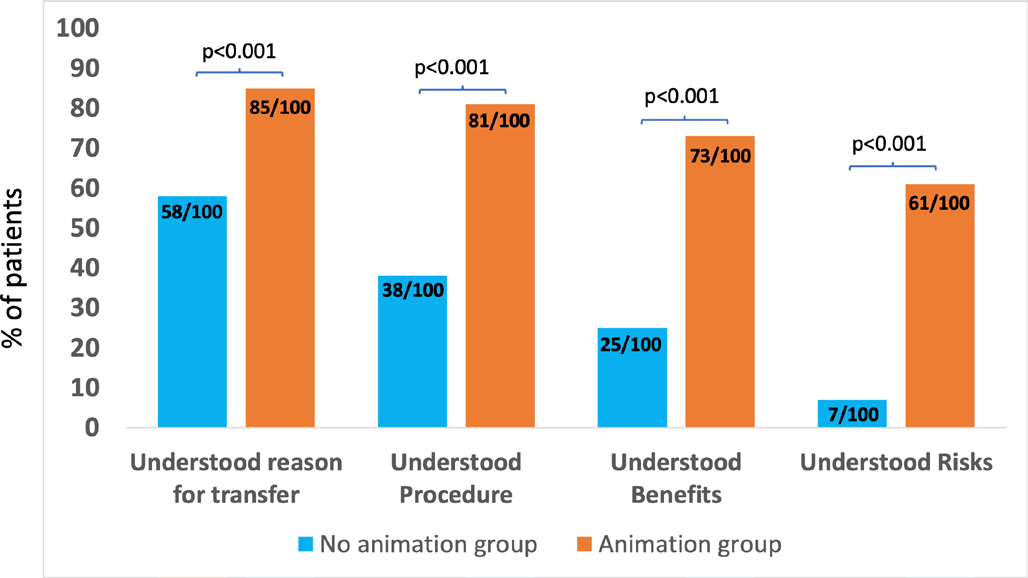
Patient-reported understanding before consent for urgent angiography and angioplasty among patients in the no animation group (n=100) and patients in the animation group (n=100).

Figure 2 compares patient-reported understanding of the reason for transfer, the procedure, its benefits and risks in the no animation group (n=100) and in the animation group (n=100), showing statistically significant differences between the groups (p<0.001 for each comparison, which remained <0.001 after correction for multiple testing). Online supplementary figure 1 shows an analysis of the 83 patients in the animation group, who watched the animation before inter-hospital transfer (3 patients declined and 14 were never offered it) compared with the no animation group, showing larger differences between groups (p<0.001 for each comparison). Among those who watched the animation, 98% of patients (81 of 83) reported it had helped their understanding of the procedure.

**Figure 3:**
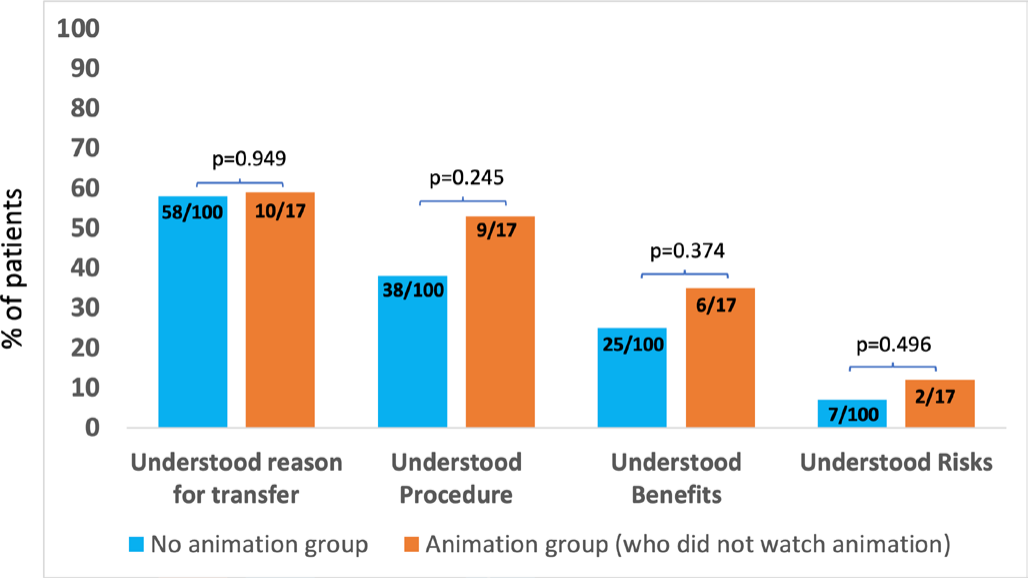
Patient-reported understanding before consent for urgent angiography and angioplasty among patients in the no animation group (n=100) and patients in the animation group who did not watch the animation (n=17).

Figure 3 compares reported understanding among the 17 patients in the animation group who did not watch the animation with that in the no animation group. The results are similar, with no statistically significant differences between the two groups. Online supplementary figure 2 compares reported understanding in the 83 patients in the animation group who watched the animation with the 17 patients who did not, showing statistically significant differences between groups (p<0.001 for all comparisons). Online supplementary figure 3 compares reported understanding in patients in the animation group, for whom animations in their native language were available (86) and for whom they were not (14) and therefore could only watch the animation in non-native English. The results were similar with no statistically significant differences in reported understanding between groups.

Table 2 gives the relative differences (animation group/no animation group) in understanding expressed as a ratio together with 95% CIs. In the animation group compared with the no animation group, there was a 1.5-fold greater understanding of the reason for transfer (1.2 to 1.8), a 2.1-fold greater understanding of the procedure (1.6 to 2.8), a 2.9-fold greater understanding of the benefits (2.0 to 4.2) and an 8.7-fold greater understanding of the risks (4.2 to 18.1).

**Table 2:** Relative understanding of angiography and angioplasty before consent in animation and no animation groups

Patient-reported understanding of	Relative understanding (ratio) animation:no animation group	95% CI	P value
Reason for transfer	1.5	1.2 to 1.8	<0.001
What procedure involves	2.1	1.6 to 2.8	<0.001
Benefits of procedure	2.9	2.0 to 4.2	<0.001
Risks of procedure	8.7	4.2 to 18.1	<0.001

## Discussion

Our results show that in patients with an acute coronary syndrome who are transferred between hospitals for urgent angiography and angioplasty, only a little over half reported understanding the reason for transfer, about 1 in 3 what the procedure involves, 1 in 4 the potential benefits and 1 in 12 the potential risks. Introduction of animations at the referring hospitals before transfer was feasible and substantially improved reported understanding before consent.

The use of images over text has been shown to increase patient understanding and recall of health information,^8 9^ and video recordings of health professionals have been used to reduce anxiety and improve patient satisfaction and comprehension before surgical procedures.^10–14^ Two recent randomised trials, one using video^15^ and the other comic strips,^16^ demonstrated improved patient comprehension and satisfaction over written information before elective coronary angiography. The present work, using multi-language animations, extends this approach to the emergency setting. The approximate ninefold increase in reported understanding of the risks exceeds the threefold improvement we previously observed in the elective setting,^5^ a difference that probably relates to the lower starting level of understanding among emergency patients, whose need for treatment was unexpected.

Almost all patients were transferred for their procedure within 72 hours of admission to the referring hospital, as recommended in practice guidelines,^17^ providing an opportunity to view the animations while waiting for transfer and allowing time for reflection before, rather than only on the day of, their procedure, as is also recommended.^3 18^ The animations do not replace a doctor’s duty to inform before consent, but help support explanation of the benefits and risks of the procedure in a consistent way, reducing variability in the quality of communication; about 80% of patients watched the animation and 98% said it helped their understanding of the procedure.

A failure to adequately inform patients about the procedures they undergo is regarded as a failure in the duty of medical care.^18 19^ Alleged failures to inform before consent are a common cause for patient complaint when complications arise. In 2018/2019, about £62 000 000 were paid by the NHS in settled claims where a failure to warn before consent was identified as the cause.^20^ Improving communication before consent is needed to improve the quality of care and is also a means of reducing cost to the NHS and other providers of healthcare. If the observed threefold (elective) to ninefold (emergency) improvement in reported understanding of procedure risks translates into a comparable reduction in claims due to a failure to understand the risks, widespread implementation of animations to support consent has the potential to substantially reduce litigation costs. Evidence from NHS Resolution,^20^ the UK department responsible for settling such claims, indicates that claim costs have increased steeply in the wake of the 2015 Montgomery judgement,^21^ so the potential exposure is likely to increase and with it, the potential benefits of broadening the use of animations to support consent.

Introducing any new initiative into a busy hospital with entrenched practices is challenging. The success of this project was helped by seeking local governance approval before implementation which was easily granted because the problem of inadequate explanation before consent was widely recognised; this initiative was seen as a worthwhile activity from the outset. Other practical steps which were applied usefully at all hospitals are summarised in box 1, together with simple strategies to improve and sustain the initiative going forward. The use of internet-free videobooks was a particular strength as many patients were unfamiliar with QR codes and internet access on wards was patchy. The QR codes, however, were helpful in engaging staff and other, often younger, family members who then shared in the understanding of the procedure and the decision to proceed with it.

Box 1 **Factors influencing implementation of animation supported consent**

**What helped implementation**

- Presenting initiative at district hospital governance meeting at outset.
- ‘Prescribing’ the animations on the consultant ward round.
- Use of videobooks on wards without internet.
- Use of weblink and QR codes on wards with internet.
- Adding animation to inter-hospital transfer checklist.
- Adding animation initiative to junior doctor induction.

**What hindered implementation**

- Patients admitted to non-cardiac wards without videobooks.
- Language barriers.

**What would improve implementation**

- Increased number of videobooks on outlying wards.
- Printing QR codes (linking to animation) on junior doctor daily patient lists.
- Increasing range of languages available.
- Periodic central audit of uptake fed back to referring hospital.

### Limitations

Not all patients awaiting inter-hospital transfer watched the animation as intended; 3 declined and 14 were never shown it, mainly because these patients were located on outlying wards which did not have access to the videobook. The level of reported understanding among these 17 patients was low and almost identical to that among the no animation group, a result that provides internal validity for the positive results in the animation group. The comparison between the no animation and animation groups was not randomised so bias is possible. However, the patients were consecutive, unselected and evenly matched in age, gender and language, so it is unlikely that such a large difference in results between the two groups is due to anything other than use of the animations. Objective assessment of understanding was not assessed; our results, like practice in general, were based on patient-reported understanding of their procedure. At the time of this service development, the animations were only available in five different languages and the population of patients spoke 14 different languages (overall about 40% were not native English speakers with similar proportions in the animation and no animation groups). Expanding translation to other languages has the potential to further improve understanding. Nonetheless, of the 14 non–English-speaking patients in the animation group who could only watch the English version (voice-overs in their languages of Somali, Romanian, Tamil, Urdu, Cantonese, French, Spanish and Arabic were not available), all but one found the animations helpful in understanding the procedure, indicating the strength of the animation, alone, in communication.

### Conclusion

Viewing multi-language animations of angiography and angioplasty before consent in the emergency clinical setting is feasible, provides time for reflection and is associated with substantially greater reported understanding of the procedures and associated risks. The approach is not limited to cardiology and has the potential to be applied to other specialties in medicine and surgery.

**Key messages**

**What is already known on this subject?**

- Patient-reported understanding at the time of consent for angiography and angioplasty is often incomplete, especially in the urgent clinical setting.
- Language barriers and time constraints are significant obstacles to effective communication.

**What this study adds?**

- Multi-language animations explaining procedures can be delivered online or using internet-free videobooks.
- Introduction of animations into practice during the 48 hours between acute presentation and inter-hospital transfer is feasible and substantially improves reported understanding of the benefits and risks of the procedure.

**How might this impact on clinical practice?**

- Routine use of animations to support consent has the potential to increase quality and reduce costs associated with consent-related claims.
- Animation supported consent is not limited to cardiac procedures and has applications across medical specialties.
