# Supplementary material for: Animation-supported consent for urgent angiography and angioplasty: a service improvement initiative
Source: Heart. 2020 Mar 10;106(22):1747–51. doi: 10.1136/heartjnl-2019-316227 (PMC7656148; doi:10.1136/heartjnl-2019-316227)
Supplement: Supplementary data [file heartjnl-2019-316227supp001.pdf]

**Supplementary Appendix**

Contents

1. List of participating District Hospitals
2. Questionnaire
3. Supplementary Figure 1
4. Supplementary Figure 2
5. Supplementary Figure 3

**List of participating District Hospitals**

Whipps Cross Hospital, Whipps Cross Rd, Leytonstone, London E11 1NR

Newham University Hospital, Glen Rd, London E13 8SL

Whittington Hospital, Magdala Ave, London N19 5NF

University College Hospital, 235 Euston Road, Bloomsbury, London, NW1 2BU

Queens Hospital, Queen's Hospital, Rom Valley Way, Romford RM7 0AG

King George Hospital, Barley Ln, Goodmayes, Ilford IG3 8YB

Royal London Hospital, Whitechapel Rd, Whitechapel, London E1 1FR

Homerton University Hospital, Homerton Row, London E9 6SR

North Middlesex University Hospital, Sterling Way, London, N18 1QX

All hospitals refer to Barts Heart Centre, W Smithfield, London EC1A 7BE for angiography and angioplasty.

Barts Health NHS
NHS Trust

# Questionnaire

**Please answer the following questions**
**to help us improve our service and the patient experience**
*Circle the response that best applies to the following statements*

| Date | | 2019 |
|---|---|---|

**1. Do you know why you have been transferred to St Bartholomew's Hospital today?**

| Yes, completely | Yes, but only partly | No |
|---|---|---|

**2. Do you understand what the procedure will involve today ?**

| Yes, completely | Yes, but only partly | No |
|---|---|---|

**3. Do you understand the possible benefits of the procedure today?**

| Yes, completely | Yes, but only partly | No |
|---|---|---|

**4. Do you understand the possible risks of the procedure today ?**

| Yes, completely | Yes, but only partly | No |
|---|---|---|

**5. Did you watch the video of the procedure before being transferred to this hospital?**

| Yes | | No |
|---|---|---|

**6. Did the video of the procedure help your understanding of the procedure?**

| Yes | | No |
|---|---|---|

**7. Was there anything in the video that was difficult to understand?**

Please comment here:

**This section is for completion by your doctor or nurse**

| | | | | | |
|---|---|---|---|---|---|
| **Procedure:** | Angio ?Proceed | | | | |
| **Referring Hospital:** | RLH | Whittington | | Newham | UCLH |
| | Queens | Whipps | KGH | Homerton | North Mid |
| **Age:** | | | | | |
| **Gender:** | | | | | |
| **English-speaker** | Yes | No | | | |
| **If No, First language:** | | | | | |

**Supplementary Figure 1:** Analysis of patient-reported understanding before consent for urgent angiography and angioplasty among patients in the no animation group (n=100) and patients in the animation group who watched the animation (n=83).

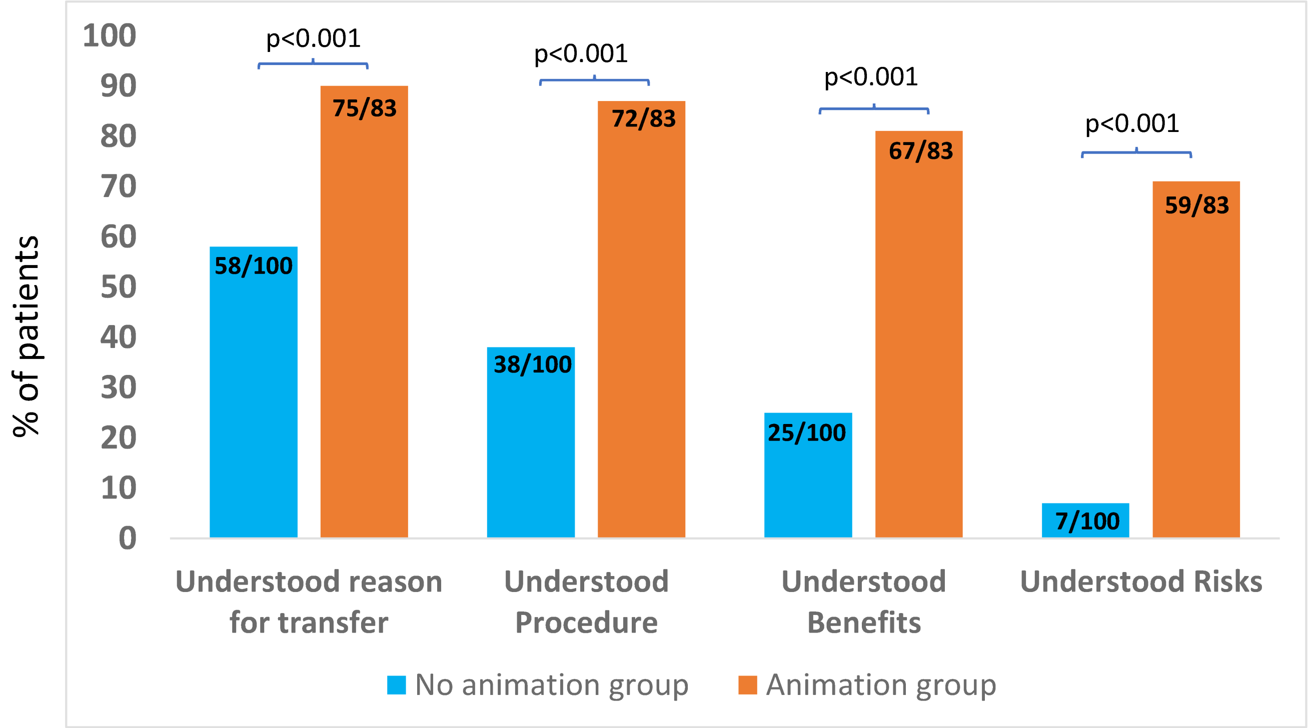

**Supplementary Figure 2:** Patient-reported understanding before consent for urgent angiography and angioplasty among patients in the animation group who did not watch the animation (n=17) and those who did (n=83).

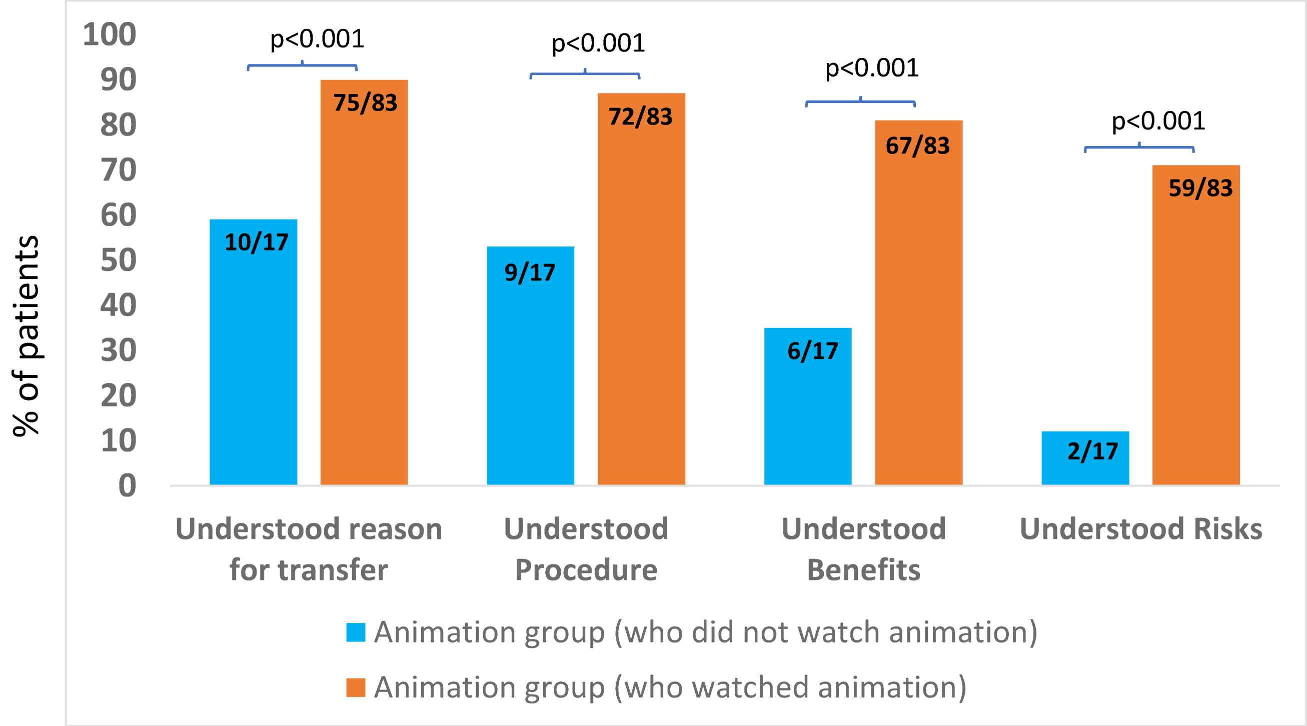

**Supplementary Figure 3:** Patient-reported understanding before consent for urgent angiography and angioplasty among patients in the animation group for whom native language animations were available (86) and for whom they were not (14*) who could only watch in non-native English.

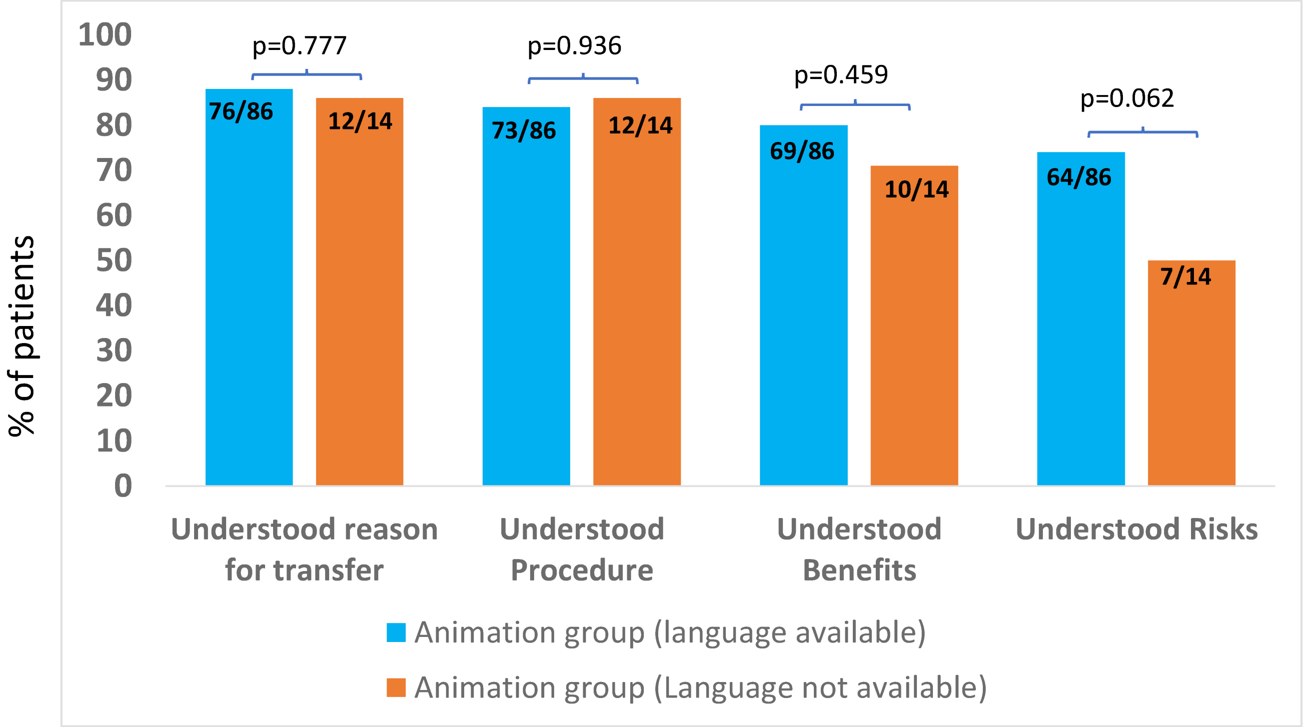


* 13/14 watched the animation in English (One Cantonese patient declined)
